# Non-pharmacological treatment for depressed older patients in primary care: A systematic review and meta-analysis

**DOI:** 10.1371/journal.pone.0184666

**Published:** 2017-09-22

**Authors:** Floor Holvast, Btissame Massoudi, Richard C. Oude Voshaar, Peter F. M. Verhaak

**Affiliations:** 1 University of Groningen, University Medical Center Groningen, Department of General Practice, Groningen, the Netherlands; 2 University of Groningen, University Medical Center Groningen, University Center of Psychiatry, Groningen, The Netherlands; 3 NIVEL, Netherlands Institute of Health Services Research, Utrecht, the Netherlands; Universidade Federal do Rio de Janeiro, BRAZIL

## Abstract

**Background:**

Late-life depression is most often treated in primary care, and it usually coincides with chronic somatic diseases. Given that antidepressants contribute to polypharmacy in these patients, and potentially to interactions with other drugs, non-pharmacological treatments are essential. In this systematic review and meta-analysis, we aimed to present an overview of the non-pharmacological treatments available in primary care for late-life depression.

**Method:**

The databases of PubMed, PsychINFO, and the Cochrane Central Register of Controlled Trials were systematically searched in January 2017 with combinations of MeSH-terms and free text words for “general practice,” “older adults,” “depression,” and “non-pharmacological treatment”. All studies with empirical data concerning adults aged 60 years or older were included, and the results were stratified by primary care, and community setting. We narratively reviewed the results and performed a meta-analysis on cognitive behavioral therapy in the primary care setting.

**Results:**

We included 11 studies conducted in primary care, which covered the following five treatment modalities: cognitive behavioral therapy, exercise, problem-solving therapy, behavioral activation, and bright-light therapy. Overall, the meta-analysis showed a small effect for cognitive behavioral therapy, with one study also showing that bright-light therapy was effective. Another 18 studies, which evaluated potential non-pharmacological interventions in the community suitable for implementation, indicated that bibliotherapy, life-review, problem-solving therapy, and cognitive behavioral therapy were effective at short-term follow-up.

**Discussion:**

We conclude that the effects of several treatments are promising, but need to be replicated before they can be implemented more widely in primary care. Although more treatment modalities were effective in a community setting, more research is needed to investigate whether these treatments are also applicable in primary care.

**Trial registration:**

PROSPERO CRD42016038442.

## Introduction

Depression is a common disorder among older adults, with an estimated one-year prevalence of 10% in primary care [1,2]. These older patients are most often treated in primary care [3], and only a few are referred to specialist mental healthcare services [4,5]. This is consistent with research indicating that older adults prefer to consult their general practitioner for mental health problems [6,7]. If depression is treated, most of these patients will be treated with an antidepressant [4]. However, depression in older adults often co-occurs with chronic somatic disease [8] and in the context of polypharmacy [9]. Prescribing antidepressants therefore increases the risk of adverse drug-related events [10], as evidenced by the fact that two-thirds of elderly antidepressant users receive drugs that are either contraindicated or have the potential for moderate to major interactions [11,12]. Moreover, tricyclic antidepressants, and to a minor degree newer agents like SSRIs, often have anticholinergic and sedative effects that are associated with physical and cognitive impairment [13–15].

Evidence-based non-pharmacological treatment options are needed for the treatment of depression in older adults, particularly in primary care. Despite this, the most recent systematic review focusing on the treatment for late-life depression in primary care was performed more than 15 years ago [16]. Although other systematic reviews and meta-analyses focusing on the psychological treatment of late-life depression have concluded that psychological therapies seem effective [17–20], these had limitations precluding the generalization of their results to primary care settings. First of all, all reviews included studies conducted in clinical settings. Moreover, the most recently conducted review included only six RCTs. Three of these six RCTs were conducted in a primary care setting, with even two of them relying on an academic team to provide the intervention at home [18]. Furthermore, two previously conducted reviews also included middle-aged adults (50+) [17,19]. Since, depression may be more heterogeneous in primary care, and treatment may be less structured, this precludes generalizability of the results of these previous systematic reviews to primary care. Given that primary care is the predominant setting in which depression in older adults is treated, it is essential that an up-to-date summary is available to inform practitioners of the evidence base for non-pharmacological treatments in this setting.

We aimed to present an overview of the evidence for non-pharmacological treatment options for depression in older adults (60+) within primary care, to provide up-to-date, evidence-based information to inform primary care physicians about possible alternatives for antidepressant treatment with its side-effects, interactions and contribution to polypharmacy.

## Methods

### Search strategy

The protocol for this systematic review was registered at PROSPERO (CRD42016038442). We performed an extensive search in the databases of PubMed, PsychINFO, and the Cochrane Central Register of Controlled Trials. We used the following search terms: (general practice OR synonym) AND (depressive disorder OR synonym) AND (aged OR synonym) AND (non-pharmacological treatment OR synonym). Free text words and index terms were used (MeSH for PubMed and Thesaurus for PsychINFO). We searched for articles until the January 2^nd^, 2017. The full search strategies for the three databases are presented in S1 Appendix.

### Identification and selection of studies

To be as comprehensive as possible, we decided not to restrict the searches to randomized controlled trials (RCTs). Results of other study types (e.g. cohort studies) were used to identify promising therapeutic strategies subject to future research. Therefore, we included all empirical studies that met the following criteria: (a) sample sizes ≥5 patients; (b) depression as the primary outcome; (c) a study population of adults aged ≥60 years at the moment of inclusion (or there were adequately reported sub-analyses of adults ≥60 years); (d) was conducted in a primary care or community setting; and (e) reported non-pharmacological treatments applicable in these settings. We set no language or date restrictions.

Depression was defined as either an identified depressive disorder according to DSM or ICD criteria determined by a validated diagnostic interview or instrument, or as an elevated score on a screening tool. Since there is no known golden standard for the identification of depression in later life, we decided to include all studies focusing on depression, regardless of their depression inclusion criterion. The age cut-off of 60 was used, because this is the mostly used cut-off for late-onset depression [21]. In addition, the earlier review regarding treatment of depression in primary care [16] also included studies focusing on adults aged 60+.

Studies were excluded if they met the following criteria: (a) included bipolar disorder, psychotic depression, or depression with suicide ideation, which are considered indicative for referral to secondline treatment [22]; (b) focused on caregivers instead of patients; (c) studied the effect of a non-pharmacological intervention as an adjunct to pharmacotherapy; (d) studied the effect of service-level intervention, such as collaborative or stepped care; or (e) studied the effect of an intervention to prevent depression.

Studies were independently screened and selected for inclusion by two authors (FH and BM). First, titles were screened to exclude irrelevant papers, and the remaining abstracts were then scrutinized in detail. Of the potentially relevant papers, full texts were retrieved to determine whether the inclusion criteria were met. In cases of disagreement, consensus was reached based on discussion and, if necessary, consultation with a third author (PV). We searched for additional articles by studying published study protocols lacking published follow-up data, by checking the reference lists of the included publications and of relevant systematic reviews and meta-analyses [17–20], and by screening conference abstracts. If necessary, corresponding authors of possible relevant papers were contacted.

### Analysis

#### Quality assessment

The Cochrane risk of bias tool was used for the quality assessment of included RCTs [23]. This was done independently by two authors (FH and BM). Studies were not excluded based on the quality assessment, but the quality was considered when comparing the different studies, when interpreting the results, and when recommendations for future studies were formulated.

#### Data extraction

Two authors (FH and BM) independently extracted data from all included studies. The following data were extracted: year of study, study design, sample size, setting (primary care, community), population characteristics (age, gender, comorbidity), treatment type and characteristics (e.g. individual/group, number of sessions), diagnosis at baseline and diagnostic tool, main result, percentage that declined participation, duration of follow-up, percentage lost to follow-up, and percentage that adhered to treatment. Included studies were classified to the setting in which they were conducted, namely primary care setting or community setting. Studies were considered primary care studies if the study recruited participants in primary care and the intervention was delivered in that setting. Studies were considered community studies when participants were recruited from the community, for example, by means of self-referral.

The results were then summarized into the following three categories: (1) mean change, defined as the difference in depressive symptoms between baseline and follow-up measurement; (2) responders, defined as a ≥50% symptom reduction in the outcome measure between baseline and follow-up (unless stated otherwise); and (3) remission from depression at follow-up measurement. The definition of remission differed between studies. The mean change in depressive symptom scores was the primary outcome of this review.

#### Analysis

We narratively reviewed the included studies by type of treatment, with the results stratified by setting (primary care or community). Given that most studies in primary care have focused on the effect of cognitive behavior therapy (CBT), we chose to perform a meta-analysis for the effect of this intervention in the primary care setting. Because of expected heterogeneity of the studies, a random-effects model was used to pool the effect of CBT on depression. We calculated the standardized mean differences (SMD) using the mean scores and standard deviations immediately after treatment and at long-term follow-up for the intervention and control groups in the studies included in the meta-analysis. If these data were not available in the original articles, they were calculated by the researchers, using the published data. If studies varied in measurement time points during long-term follow-up, we calculated the SMDs for the points closest to 6 months. Statistical heterogeneity was evaluated by the chi-square and I^2^ tests. Inter-study heterogeneity was considered significant for p < 0.1 and I^2^ > 50%. The meta-analysis was conducted using Review Manager (Version 5.3. Copenhagen: The Nordic Cochrane Centre, The Cochrane Collaboration, 2014).

## Results

### Selection of studies and characteristics of included studies

**Fig 1:**
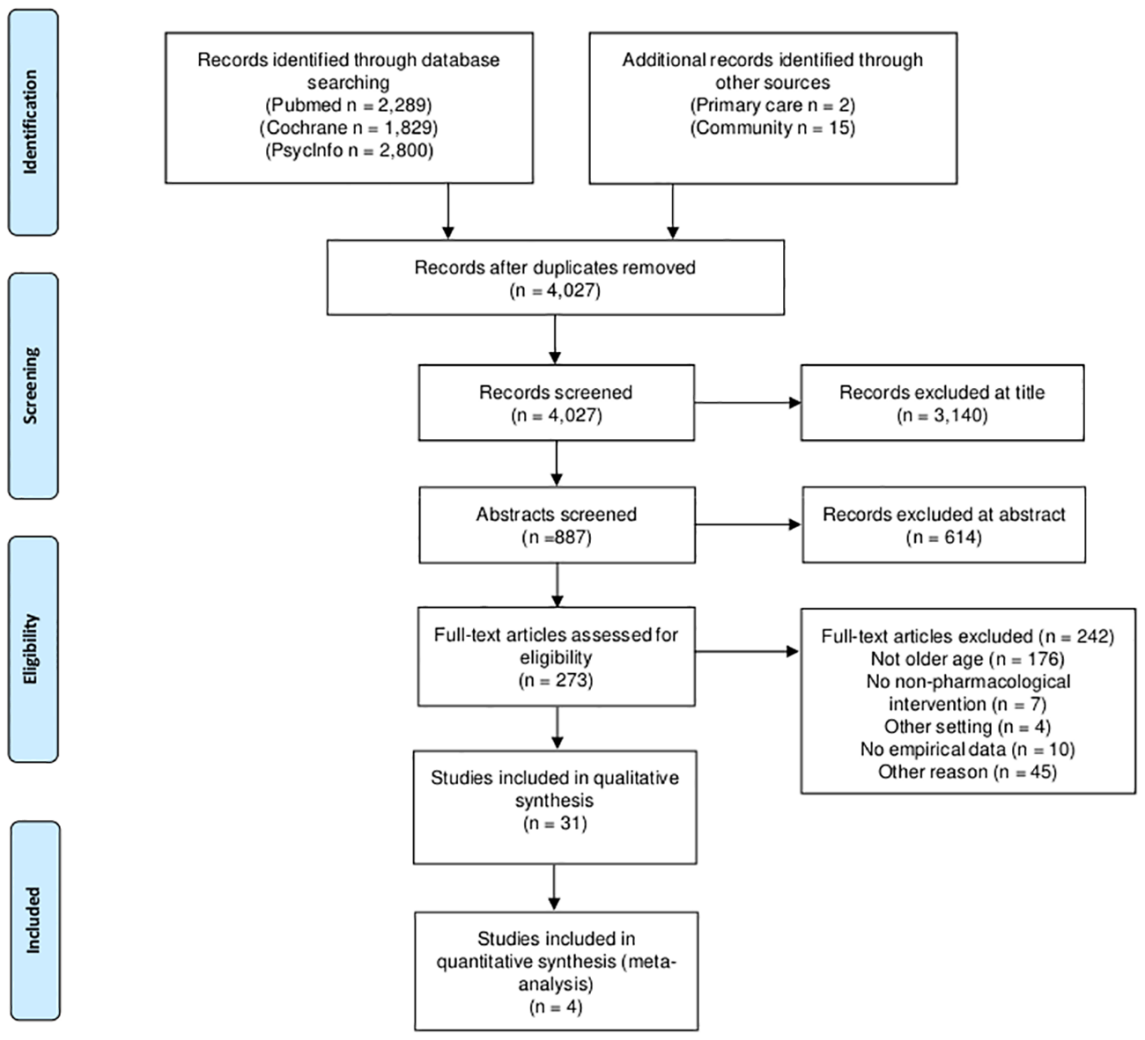
PRISMA flow diagram of study selection.

Fig 1 summarizes the process of study inclusion. In total, 4027 references were screened and 273 full text papers were retrieved, of which 17 were identified through cited reference search. Of these, 31 were included that consisted of 29 different studies (27 RCTs, two cohort studies). Of two RCTs two references of each were included, one reporting short-term follow-up and the other long-term follow-up. Eleven primary care studies and 18 community studies were included.

Table 1 shows the characteristics of the 11 included studies that were conducted in primary care settings (10 RCTs, 1529 patients; 1 cohort study, 14 patients). The interventions studied included CBT (n = 5), exercise (n = 2), problem-solving therapy (PST; n = 1), a combination of CBT and bibliotherapy (n = 1), behavioral activation (BA; n = 1), and bright-light therapy (n = 1). Follow-up ranged from 1 week up to 12 months.

**Table 1:** Characteristics of studies conducted in primary care.

Study (year)	Design	Setting (country)	Diagnostic inclusion criterion	Intervention	N	Control (if applicable)	N	Mode of therapy	Age mean (min)	Female (%)	Antidepressant therapy	Specific baseline characteristics	Declined participation (%)	Follow-up period	Loss to follow-up (%)	Adherence (%)
Arean (2005) [24]	RCT	Primary care and community (USA)	Major depression and dysthymia (SCID)	CBT	20	1.CCM; 2.CCM + CBT	1. 27; 2. 25	Group therapy, by psychologist or social worker, 18 sessions/ week for 16 weeks, 2 h each	65.3 (60+)	64.2%	AD use was an exclusion criterion, and 11 (22%) started Ads immediately after treatment	≤$15,000 household income	14%	Post-treatment 6 months 12 months	30.6% 38.9% 33.3%	(Mean session attendance) CBT 9.8 CCM 13.9 CBT + CCM 9.1
Garcia-Pena (2015) [25]	RCT	Primary care (Mexico)	Depressive symptoms (PHQ-9 cut-off 2–6)	CBT	41	CAU by GP	41	Group therapy by a nurse with 10/group; 12 1.5 h sessions/week	70.8 (60+)	83%	Not reported	–	Not reported	12 weeks	1.2%	Not reported
Gum (2016) [26]	Pilot Cohort	Primary care (USA)	Mild to moderate depressive symptoms (PHQ-9 cut-off 5–14)	Behavioral activation	14	n.a.	n.a.	Individual, 1 session, 90 minutes, by psychologist, identifying life values, select activities, and establish weekly goals. FU by 3 phone calls	70.2 (60+)	71.4%	Baseline: 35.7% psychotropic medication	–	47.8%	4 weeks	33.3%	Not reported
Joling (2011) [27]	RCT	Primary care (Netherlands)	Subthreshold depression (CES-D cut-off 2–6)	CBT-based bibliotherapy	86	CAU	84	Individual therapy, 3 nurse visits max 1 h, 2 phone calls	81.5 (75+)	73.5%	Not reported	–	15.4%	2 months	14.1%	41% completed full intervention
Laidlaw (2008) [28]	RCT	Primary care (Scotland, UK)	MDD (SADS-L; HDRS ≥7<24; BDI-II ≥13<28)	CBT	21	CAU by GP	23	Individual, by psychologist, mean 8 sessions (range 2–17)	74 (60+)	65.9%	2 CBT participants on AD. CAU could include AD	–	24.3%	Post-treatment; 3 months; 6 months	13.6%; 22.7%; 43.2%	Not reported
Lamers (2010) [29]	RCT	Primary care (Netherlands)	MDD (mild/mod), minor depression, dysthymia (MINI)	Self-management + CBT	183	CAU	178	Individual therapy, by nurse, 2–10 sessions during 3 months (mean 4), 1 h each	70.7 (60+)	46.5%	AD exclusion criterion. CAU could include AD: 7 participants started during follow-up	165 DM and 176 COPD patients	Not reported	1 week 3 months; 9 months	26.9%; 33.5%; 33.2%	No difference in dropout rate between IG and CG
Lieverse (2011) [30]	RCT	Outpatient clinics and case-finding via GP offices (Netherlands)	Major depression (SCID)	Bright-light therapy	42	Dim red light	47	Individual, 60 min during early morning, for 3 week	69.3 (60+)	65.2%	Randomization stratified for AD. IG 33%; CG 38%	–	11.7%	3 weeks; 6 weeks	5.6%; 16.9%	Not clearly reported
Serfaty (2009) [31]	RCT	Primary care (UK)	Depressive disorder (Geriatric Mental State and History and Etiology Schedule; BDI-II ≥14)	CBT + CAU	70	1. Talking control + CAU; 2. CAU	1. 67; 2. 67	Individual therapy, by psychologist, max 12 sessions, 50 min each. As adjunct: “Feeling Good” handbook	74.1 (65+)	79.4%	Inclusion: stable dose for ≥8 weeks.Baseline: IG 25.75% TC 23.8% CAU 37.3% CAU could include AD	–	19.9%	4 months; 10 months	13.2%; 18.1%	Mean number of session attended was 7
Sims (2006) [32]	Pilot RCT	Primary care (Australia)	Depressive symptoms (GDS cut-off ≥11)	Progressive Resistance Training	14	Advice	18	Group / individual not reported, 3/week for 10 weeks (max. 30 sessions) + weekly phone monitoring	74.3 (65+)	65.6%	AD exclusion criterion	–	Not reported	10 weeks; 6 months	15.8%	58% met adherence criterion of 60%
Singh (2005) [33]	RCT	Primary care (Australia)	Major/minor depression, dysthymia (instrument not reported; GDS cut-off ≥14)	1. High intensity training; 2. Low intensity training	1. 20; 2. 20	CAU by GP	20	Groups of 1–8 participants, 3/week during 8 weeks, sessions of 60min. HIGH: 80% of max load. LOW: 20% of max load	69.3 (60+)	55%	Inclusion: No AD prescription within last 3 months	–	Not reported	8 weeks	10.0%	HIGH: 95%–100%; LOW: 99%–100%
Williams (2000) [34]	RCT	Primary care (USA)	Minor depression and dysthymia (PRIME-MD)	PST	138	1. placebo; 2. paroxetine	1. 140 2. 137	Individual, by psychologist, social worker, or counselor.6 sessions for 11 week (first for 1 h then 30 min each)	71 (60+)	41.5%	AD prohibited in PST	–	3.9%	11 weeks	25.1%	81,4% attended ≥4 sessions; 74.9% completed all sessions

AD, Antidepressant; BDI, Beck Depression Inventory; CAU, Care as Usual; CBT, Cognitive Behavior Therapy; CCM, Clinical Case-Management; CES-D, Center for Epidemiologic Studies Depression Scale; CG, Control Group; COPD, Chronic Obstructive Pulmonary Disease; DM, Diabetes Mellitus; FU, follow-up; GDS, Geriatric Depression Scale; GP, General Practitioner; HDRS, Hamilton Depression Rating Scale; IG, Intervention Group; MDD, Major Depressive Disorder; MINI, Mini International Neuropsychiatric Interview; n.a., not applicable; PHQ-9, Patient Health Questionnaire; RCT, Randomized Controlled Trial; SCID, Structured Clinical Interview for DSM Disorders; SADS-L, Schedule for Affective Disorders and Schizophrenia–Lifetime version; UK, United Kingdom; USA, United States of America.

Table 2 shows the characteristics of the 18 studies recruiting in the community, most of which depended on self-referral by participants (17 RCTs, 1041 patients; 1 cohort study, 22 patients). The studied treatment modalities were CBT (n = 3), bibliotherapy (n = 4), life-review (n = 3), exercise (n = 4), PST (n = 3), and receiving postcards (n = 1). In addition, 1 study compared cognitive therapy, behavioral therapy, and brief psychodynamic therapy with patients on a waiting list. Follow-up period ranged from 4 weeks to 2 years.

**Table 2:** Characteristics of included studies conducted in community settings.

Study (year)	Design	Setting (country)	Diagnostic inclusion criterion	Intervention	N	Control (if applicable)	N	Mode of therapy	Age mean (min)	Female (%)	Antidepressant therapy	Specific baseline characteristics	Declined participation (%)	Follow-up period	Loss to follow-up (%)	Adherence (%)
Chan (2013) [35]	RCT	Community (Singapore)	Mild to moderate depressive symptoms (GDS cut-off ≥4)	Life-review	14	None	12	Creating a life-story book, including personal photos. 5 sessions, 30–45min each	69.7 (60+)	80.8%	Not reported	Sampling through researcher personal network	20.7%	8 weeks	0%	Not reported
Ciechanowski (2004) [36]	RCT	Community senior service agencies (USA)	Minor depression and dysthymia (SCID)	PST+ Social activities+ Moderate physical activity	72	CAU	66	Individual, by social worker, 8 sessions during 19 week, 50 min. Followed by brief phone contact	73 (60+)	79%	Baseline: IG 40%; CG 30% During study: started AD IG 7; CG 4 Stopped AD 5 IG; 5 CG 8 dosage adjusted	–	8%	6 months; 12 months	5.1%; 8.0%	Median: 8.0 visits
Floyd (2004, 2006) [37,38]	RCT	Community (USA)	Minor and major depression, dysthymia (HRDS cut-off ≥10)	1.Bibliotherapy; 2.Cognitive psychotherapy	1. 16 2. 16	Waiting list (4 weeks)	14	1.Book “Feeling Good,” read + homework exercises, <1month. Weekly phone calls. 2.Individual, by clinical psychology graduate students, 12–20 sessions, 1–2/week	68.0 (60+)	76.1%	Inclusion: stable dose for ≥3 months Baseline: 26%	Self-referral	12.6%	Post-treatment; 3 months (IG only); 2 years (IG only)	30.4%; 43.5%; 25.8% (eligible N = 31)	Bib: Average 254 pages read. CP: 80.2% of homework assignments completed
Huang (2015) [39]	RCT	Community (Taiwan)	Depressive symptoms (GDS cut-off ≥5)	1.Exercise (PFE); 2.CBT	1. 19 2. 18	CAU	20	1.Group therapy, 2–4 per group, 3/week during 12 weeks, 50min per session, by fitness instructor. Goal: 150min/week. 2.Group therapy, 3–5 per group, 12 weekly sessions, 60–80min each, by geriatric nurse	76.5 (65+)	52.6%	No AD at inclusion or starting AD during follow-up	–	35.8%	Post-treatment; 3 months; 6 months	0%	PFE goal achievement post-treatment 100%; 3 months 63.2%; 6 months 47.4%. CBT: unknown
Imai (2015) [40]	RCT	Community (Japan)	Depressive symptoms (GDS cut-off ≥4)	Receiving postcards	93	None	91	Receiving postcards, 1/month during 8 months. Handwritten message + computer printed general message	81 (65+)	73.4%	Baseline: IG 8.9%; CG 8.3% No restrictions regarding treatment outside trial	“Social isolation” defined by eating meals alone	54.8%	12–14 months	20.7%	Not applicable
Kiosses (2010) [41]	RCT	Community (USA)	MDD (SCID, HAM-D cut-off ≥17)	Problem Adaptation Therapy	15	Supportive therapy	15	PST modified for cognitive impaired. Individual, during 12 weeks, home-delivered, by therapist	79.4 (65+)	70.0%	Inclusion: Psychotropic medication stable dose for ≥8 weeks. Baseline: 78.7% on AD in both groups	Cognitive impairment deficit (DRS≤30) & impairment iADL≥1 Self-referral	13.5%	6 weeks; 12 weeks (post-treatment)	10.0%; 16.7%	Not reported
Kiosses (2015 [42])	RCT	Community agencies (USA)	Major depression (SCID)	Problem Adaptation Therapy	37	Supportive therapy cognitive impairment	37	Problem-solving approach, individual, by psychologist / social worker / MD. 12 sessions 1/week	80.9 (65+)	74.3%	Inclusion: stable dose for ≥6 weeks. Baseline: IG 65%; CG 62%	At least mild cognitive deficit (DRS≤7) & impairment iADL≥1	Not reported	12 weeks	14.9%	Not reported
McNeil (1991) [43]	RCT	Community (Canada)	Moderate depression (BDI cut-off 12–24)	Exercise	? (total 30)	1.Social contact control; 2. Waiting list (6 weeks)	? (total 30)	Walking at vigorous pace, 3x/week, 20–40min, during 6 weeks. 2x/week accompanied with undergraduate psychologist	72.5 (?)	?	Not reported	–	Not reported	10 weeks	0%	Not reported
Moss (2012) [44]	RCT	Community (USA)	Depressive symptoms (GDS cut-off ≥5)	Behavioral Activation Bibliotherapy	13	Waiting list (4weeks)	13	Individual, self-study in workbook, weekly phone calls	77.5 (65+)	76.9%	Inclusion: stable dose for ≥1 month	–	5.5%	8 weeks	30.8%	69% completed full treatment program
Preschl (2012) [45]	RCT	Community (Switzerland)	Subsyndromic and moderate depression (BDI cut-off 10–28)	Life-review	21	Waiting list (6 weeks)	19	Individual, by psychologist, 6 sessions, 1/week during 6 weeks. Face to face and computer intervention	70 (65+)	66.7%	Baseline: IG 28.6% CG 41.2%	Self-referral	Not reported	6 weeks; 3 months (IG only)	10.0%; 33.3%	Not reported
Rosenberg (2010) [46]	Pilot Cohort	Senior community center / retirement communities (USA)	Subsyndromic depression (MINI)	Exercise (Nintendo Wii gaming)	22	n.a.	n.a.	Group / individual not reported first by physical trainer, subsequent by staff members, 3x 35min each week, during 12 weeks	78.7 (60+)	68.4%	AD exclusion criterion	–	Not reported	12 weeks; 24 weeks	13.6%; 22.7%	84% adherence of total possible days
Scogin (1987) [47]	RCT	Community (USA)	Mild to moderate depression (HRSD cut-off ≥10)	1.Cognitive bibliotherapy; 2.Control bibliotherapy (Attention Control)	1. 10 2. 8	Waiting list (4 weeks)	11	1.Book “Feeling good,” read <1month, weekly phone calls 2.Book “Man”s search for meaning,” weekly phone calls	71.0 (60+)	79.3%	Baseline: 20.7% psychotropic med. CB 33.3% AC 25.0% Waiting list 9.1%	Self-referral	Not reported	Post-treatment; 1 month (IG only)	10.3%; 31.0%	2 did not start a book, 3 did not complete a book
Scogin (1989, 1990) [48,49]	RCT	Community (USA)	Mild to moderate depression (HRSD cut-off ≥10)	1.Behavioral bibliotherapy; 2.Cognitive bibliotherapy	1. 23 2. 22	Waiting list (4 weeks)	22	1.Book “Control Your Depression,” read <1month, weekly phone calls. 2.Book “Feeling Good,” read <1month, weekly phone calls	68.3 (60+)	85.1%	Inclusion: stabilized on psychotropics. Baseline: 34.3%	Self-referral	Not reported	Post-treatment; 6 month (IG only); 2 years (IG only)	7.5%; 34.3%; 31.8% (eligible N = 44)	Both conditions: average of 85% of book was read
Serrano (2004) [50]	RCT	Community (Spain)	Clinically significant depressive symptoms (CES-D cut-off ≥16)	Life-review	25	CAU (by social services)	25	Individual, by therapist, 4 sessions, 1/week	77.2 (65+)	76.7%	AD exclusion criterion	Self-referral	18.4%	8 weeks	14%	Not reported
Singh (1997) [51]	RCT	Community	Mild depressive symptoms (BDI cut-off >12)	Exercise	17	Health education program	15	Group (1–8 individuals), high intensity progressive resistance training, during 10 weeks, 3 days/week, 45 min per session, by principal investigator	71 (60+)	62.5%	Exclusion if on AD within last 3 months	Self-referral	Not reported	10 weeks	0%	IG: 93%. CG: 95%
Thompson (1987) [52]	RCT	Community (USA)	Major depression (Research Diagnostic Criteria) BDI cut-off ≥17; HRSD cut-off ≥14	1.Cognitive therapy; 2.Behavioral therapy; 3.Brief psychodynamic therapy	1. 27 2. 25 3. 24	Waiting list (6 weeks)	20	All 3 interventions: Individual therapy, by psychologist, 16–20 sessions, 1–2/week	67.0 (60+)	67.4%	Inclusion: stabilized for ≥3 month. Baseline: % unclear	Self-referral	Not reported	Mid-treatment;Post-treatment (IG only)	Unclear	Not reported
Titov^a^ (2015) [53]	RCT	Community (Australia)	Depressive feelings	iCBT	29	Waiting list (8 weeks)	25	Individual, 5 lessons during 8 weeks + weekly contact with therapist by phone or e-mail	65 (60+)	74.1%	Not reported	Self-referral	Not reported	Post-treatment; 3 months (IG only); 12 months (IG only)	13.0%; 31.0%; 34.5%	70% completed treatment within the 8 wk course
Wuthrich (2013) [54]	RCT	Community (Australia)	DSM-IV (sub)clinical criteria for both anxiety and mood disorder (ADIS ≥3)	CBT	27	Waiting list (12 weeks)	35	Group therapy, 12 weekly sessions, 2 h each, 6–8 participants per group, by psychologist + homework	67.4 (60+)	64.5%	Baseline: 21.0% on psychotropic medication. IG 22.2 CG 20.0%. Participants were asked not to change medication during trial	Comorbid anxiety disorder. Self-referral	Not reported	Post-treatment; 3 months (IG only)	24.2%; 25.9%	Mean number of sessions attended 9.3

AD, Antidepressant; AC, Attention Control; ADIS, Anxiety Disorder Interview Schedule; BDI, Beck Depression Inventory; Bib, Bibliotherapy; CB, Cognitive bibliotherapy; CBT, Cognitive Behavior Therapy; CES-D, Center for Epidemiologic Studies Depression Scale; CG, Control Group; CP, Cognitive Psychotherapy; DRS, Dementia Rating Scale; GDS, Geriatric Depression Scale; iADL, instrumental Activities of Daily Life; HAM-D Hamilton Rating Scale for Depression; HRSD, Hamilton Rating Scale for Depression; iCBT, Individual Cognitive Behavior Therapy; IG, Intervention Group; MINI, International Neuropsychiatric Interview; PFE, Physical Fitness Exercise; PST, Problem-Solving Therapy; RCT, Randomized Controlled Trial; SCID, Structured Clinical Interview for DSM Disorders; USA, United States of America.

### Outcome data of primary care studies

**Fig 2:**
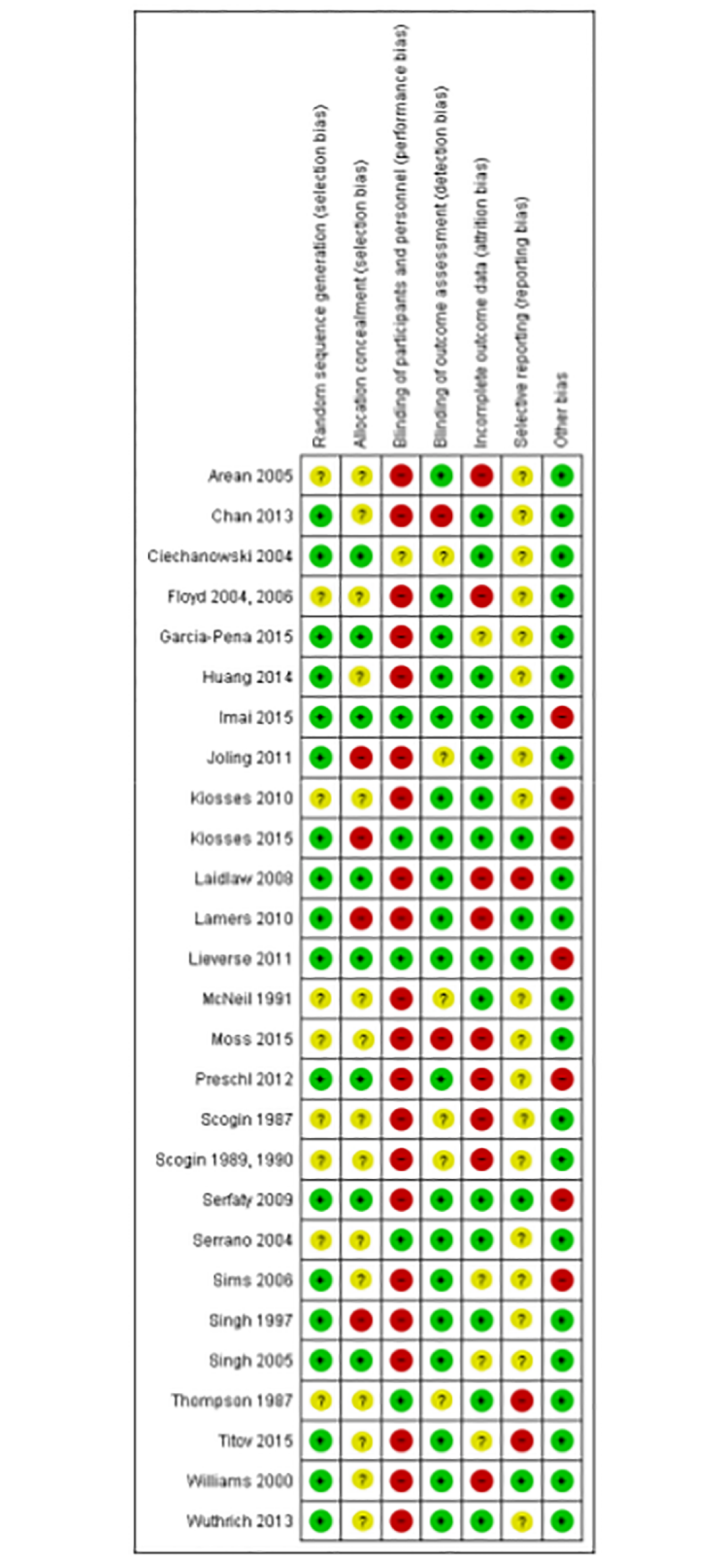
Risk of bias assessment for the included randomized controlled trials. Based on the Cochrane Collaboration’s tool for assessing risk of bias, + indicates low risk of bias,—indicates high risk of bias, and? indicates unclear risk of bias.

**Fig 3:**
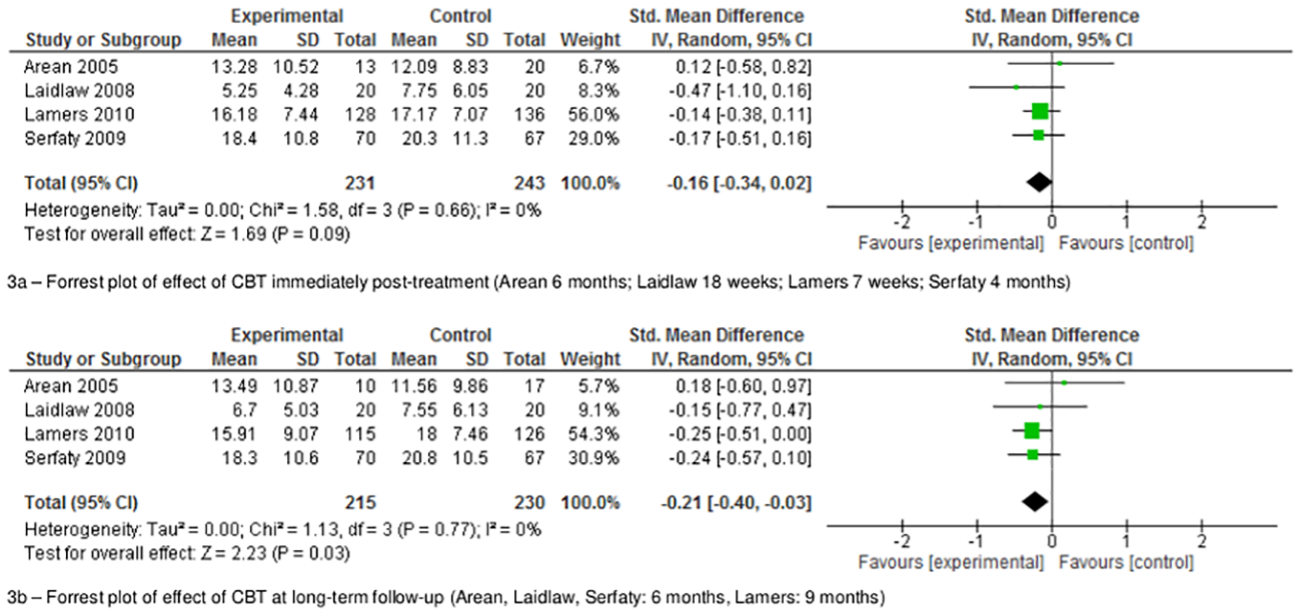
Forrest plot of the meta-analysis for studies of cognitive behavioral therapy in primary care. Control condition entered in meta-analyses specified by study: Arean (2005) [24] used clinical case-management; but, Laidlaw (2008) [28], Lamers (2010) [29], Serfaty (2009) [31] used care as usual. One of the two reported outcome measurements by Laidlaw was used in the meta-analyses, namely HRSD.

The outcome data for studies conducted in a primary care setting are presented in Table 3, and the quality assessments with corresponding scores on the subscales are presented in Fig 2. The results of the meta-analysis are summarized in Fig 3.

**Table 3:** Outcomes by intervention and control groups (if applicable) for studies conducted in primary care.

Study (year)	Treatment	Outcome measure	Follow-up	Mean change*	Responders**	Remission
Arean (2005) [24]	1.CBT; 2.CCM; 3.CBT+CCM	HDRS	Post-treatment (6 months); 6 months FU; 12 months FU	CBT -1.71 CCM -3.84 CBT+CCM -2.77; n.s.; CBT -1.50 CCM -4.37 CBT+CCM -4.81; n.s.; CBT +1.97 CCM -5.10 CBT+CCM -8.49; CBT vs other p < .01	Not reported	Not reported
Garcia-Pena (2015) [25]	CBT	PHQ-9	12 weeks FU	Not reported	IG 56.1% and CG 30%; n.s.^a^	Not reported
Gum (2016) [26]	Behavioral Activation	PHQ-9	4 weeks FU	-4.28; p = .002	Not reported	57.1%^b^
Joling (2011) [27]	CBT-based bibliotherapy	CES-D	2 months FU	IG: -4.57 and CG -4.78; p = .73	IG 46.9% and CG 43.6; p = .70^c^	IG 36.4% and CG 30%; p = .46^d^
Laidlaw (2008) [28]	CBT	HRSD BDI-II	Post-treatment (18 weeks); 3 months FU; 6 months FU	HRDS: IG -6.15 and CG -4.05; p = .15; BDI-II: IG -10.2 and CG -6.25; p = .21; HRDS: IG -6.25 and CG -5.1; p = .38; BDI-II: IG -10.6 and CG -6.6; p = .17; HRDS: IG -4.7 and CG -4.25; p = .63; BDI-II: IG -9.05 and CG -4.4; p = .18	Not reported	IG 70% and CG 40%; p.06^e^; IG 80% and CG 50%; p = .047^e^; IG 55% and CG 40%; p = .34^e^
Lamers (2010) [29]	Self-management + CBT	BDI	1 week FU; 3 months FU; 9 months FU	IG -0.92 and CG -0.53; p = .19; IG -1.22 and CG -0.21; p < .05; IG -1.19 and CG -0.30; p = .03	IG 6.3% and CG 7.4%; n.s.; IG 12.4% and CG 8.7%; n.s.; IG 17.5% and CG 7.3%; p = .02	Not reported
Lieverse (2011) [30]	Bright-Light therapy	HAM-D	Post-treatment (3 weeks); 6 weeks FU	IG -8.5 and CG -5.8; p = 0.03; IG -10.0 and CG -5.4; p = .001	IG 50% and CG 41%; p = .20; IG 58% and CG 34%; p = .05	Not reported
Serfaty (2009) [31]	CBT	BDI-II	4 months FU; 10 months FU	CBT -8.9 TC -6.2 CAU -7.4; CBT vs other p < .05; TC vs CAU n.s.; CBT -9.0 TC -6.1 CAU -6.9; CBT vs other p < .05; TC vs CAU n.s.	CBT 33% and TC 21% and CAU 23%; p-value not reported	Not reported
Sims (2006) [32]	Progressive Resistance Training	GDS	10 weeks FU; 6 months FU	IG -0.41 and CG -0.22; n.s.; IG -1.14 and CG -0.34; n.s.	Not reported	Not reported
Singh (2005) [33]	1. High intensity training; 2. Low intensity training	HRSD/GDS	8 weeks FU	HRSD: HIGH -9.5 LOW -7.1 GP -5.3; p = .14; GDS: HIGH -11.6 LOW -8.7 GP -4.7; p = .006	HRSD: HIGH 61% LOW 29% GP 21%; HIGH vs LOW p = .05; HIGH vs GP p < .02; LOW vs GP p = .56	Not reported
Williams (2000) [34]	PST	HSCL-D-20	Post-treatment (11 weeks)	PST -0.52 paroxetine -0.61 placebo -0.40; PST vs paroxetine p = .17; PST vs placebo p = .13	Not reported	Not reported

BDI, Beck Depression Inventory; CAU, Care as Usual; CBT, Cognitive Behavior Therapy; CCM, Clinical Case-Management; CES-D, Center for Epidemiologic Studies Depression Scale; CG, Control Group; FU, Follow-Up; GDS, Geriatric Depression Scale; HAM-D Hamilton Rating Scale for Depression; HDRS, Hamilton Depression Rating Scale; HSCL-D, Hopkins Symptom Checklist for Depression; IG, Intervention Group; n.s., not significant; PHQ-9, Patient Health Questionnaire.

* Difference between baseline measurement and follow-up measurement;

**Defined as ≥50% reduction in outcome measure unless stated otherwise;

^a^ Defined as a decrease of ≥5 points on the PHQ-9 after 12 weeks;

^b^ Defined as a PHQ-9 score ≤4;

^c^ Defined as a decrease of ≥5 points on the CES-D;

^d^ Defined as a decrease of ≥5 points or more on the CES-D and a post-test score <16;

^e^ Determined by RDC (Research Diagnostic Categorization as <4 symptoms of depression)

#### Cognitive behavioral therapy

Five studies assessed the effect of CBT on depression in older adults: three assessed the effect of CBT alone [25,28,31], one assessed its use in combination with self-management [29], and one compared CBT with clinical case-management [24]. CBT was delivered individually in three out of the five studies [28,29,31], and as a group therapy in the other two [24,25].

CBT delivered as individual therapy was more effective in reducing depressive symptoms at 4 and 12 months’ follow-up compared with both control groups (talking control and care as usual) [31]. In a study where CBT was delivered by individual therapy, CBT was not effective at reducing depressive symptoms immediately after treatment or at 3 and 6 months’ follow-up [28]. Equally, in another study where CBT was delivered by group therapy, it was no more effective in achieving response (determined by a decrease of ≥5 points in the PHQ-9 [Patient Health Questionnaire]) compared with care as usual at 12 weeks’ follow-up [25]; however, this later study did not report the mean change in depressive symptoms. In other research, clinical case-management was more effective than CBT at 12 months’ follow-up [24]. By contrast, CBT in combination with self-management was shown to reduce depressive symptoms at 3 and 9 months’ follow-up [29].

Fig 3 shows the results of the meta-analysis. Four out of the five studies focusing on CBT in primary care could be included in the meta-analysis; the fifth study could not be included because it did not report continuous baseline and follow-up data [25], and because the authors could not be reached by e-mail. The meta-analysis demonstrated that CBT had no effect on depression immediately after treatment (SMD -0.16 [-0.34–0.02], *I*^2^ = 0%, *Z* = 1.69, p = 0.09). A statistical significant effect was found at 6 months’ follow-up, but the effect size was only small (SMD -0.21 [-0.40 –-0.03], *I*^2^ = 0%, *Z* = 2.23, p = 0.03). No statistically significant heterogeneity was found between the studies (χ^2^ = 1.58 [p = 0.66] and 1.13 χ^2^ = [p = 0.77], respectively; *I*^2^ = 0% in both analyses).

To summarize, CBT was effective in two of the five studies, of which one was assessed to have the lowest risk of bias. This effect was confirmed in the meta-analysis at six months’ follow-up. The two studies demonstrating a beneficial effect of CBT used individually delivered treatment rather than group therapy.

#### Exercise

Two studies assessed the effect of exercise [32,33]. Compared to a control group receiving information about exercise and local exercise options, progressive resistance training was not more effective in reducing depressive symptoms at 10 weeks’ and 6 months’ follow-up [32]. But, high and low intensity training were both more effective in reducing depressive symptoms at 8 weeks’ follow-up based on self-reported, but not observer-rated, measures [33]. The risk of bias was assessed as moderate for both studies.

#### Other

Treatment modalities in the “other” category included PST, CBT-based bibliotherapy, behavioral activation, and bright-light therapy; all four were delivered individually. Of the two studies with a low risk of bias, bright-light therapy was effective [30], whereas PST was not [34]. In a study of moderate quality, CBT-based bibliotherapy was shown to be no more effective than care as usual [27]. Behavioral activation, which was only studied in a pilot cohort, was found to reduce symptoms of depression at 4 weeks’ follow-up [26].

### Outcome data of studies in community settings

Outcome data for studies conducted in the community are presented in Table 4, and the quality assessment with corresponding scores on the subscales is presented in Fig 2.

**Table 4:** Outcomes among intervention and control groups (if applicable) for included studies conducted in community settings.

Study (year)	Treatment	Outcome measure	Follow-up	Mean change*	Responders**	Remission
Chan (2013) [35]	Life-review	GDS	8 weeks	IG -5.4 CG -1.0; p < .001	Not reported	Not reported
Ciechanowski (2004) [36]	PST; Social activities; Moderate physical activity	HSCL-20	6 months FU; 12 months FU	IG -0.59 and CG -0.03; p < .001; IG -0.48 and CG -0.19; p = .03	IG 54% and CG 8%; P < .001; IG 43% and CG 15%; p < .001	IG 44% and CG 10%; p < .001^a^ IG 36% and CG 12%; p = .002^a^
Floyd (2004, 2006) [37,38]	1.Bibliotherapy (Bib); 2.Cognitive psychotherapy (CP)	HRSD/GDS	Post-treatment (Bib 4 weeks; CP 12 weeks); 3 months FU (IG only); 2 years FU (IG combined)	HRSD: B -6.81 CP -10.62 CG -0.29; GDS B -5.6 CP -11.68 CG -0.79; B vs CG, CP vs CG, B vs CP all p < .05; HRSD: B -12.56 CP -6.22; GDS: B -8.46CP -10.06; HRSD: further improvement for B p < .05; CP n.s.; GDS: no change compared with post-treatment for B and CP; HRSD: -10.87; GDS: -8.73; HRDS and GDS no change compared with post-treatment	Not reported	B 35%; CP 57%; n.s.^b^
Huang (2015) [39]	1.Exercise (PFE); 2. CBT	GDS	Post-treatment (3 months FU); 3 months FU; 6 months FU	PFE -4.0 CBT -3.5 CG -2.0; p = .012; PFE -4.21 CBT 2.61 CG -2.45; p = .12; PFE -3.84 CBT -3.0 CG -2.1; p = .20	Not reported	PFE 57.9% CBT 61.1% CG 30%^c^^;^ PFE 68.4% CBT 61.1% CG 45%^c^; PFE 63.2% CBT 66.7% CG 35%^c^
Imai (2015) [40]	Receiving postcards	GDS	12–14 months FU	IG 0.5 and CG 0.7; n.s.	Not reported	Not reported
Kiosses (2010) [41]	Problem Adaptation Therapy	HAM-D	6 weeks FU; 12 weeks FU	IG -11.33 and CG -7.65; IG -13.48 and CG 8.65; p = .03	Not reported	Not reported
Kiosses (2015) [42]	Problem Adaptation Therapy	MADRS	12 weeks FU	Baseline scores IG 21.08; CG 21.41; p = .58; IG lower scores at week 12; p = .001 (no mean difference reported)	IG 66.7% and CG 32.3%; p = .007	IG 37.8% and CG 13.5%; p.02^d^
McNeil (1991) [43]	Exercise	BDI	10 weeks FU	IG: -5.5; Attention Control -4.2; CG -0.5; IG vs Attention Control p > .05; IG vs CG p < .05; Attention Control vs CG p < .05	Not reported	Not reported
Moss (2012) [44]	Behavioral Activation Bibliotherapy	HRSD	Post-treatment (4 weeks)	IG -5.77 and CG -1.15; p = .004	Not reported	Not reported
Preschl (2012) [45]	Life-review	BDI	Post-treatment (8 weeks); 3 months FU (IG only)	IG -9.0 and CG -1.4; p < .01; IG -10.3; p < .01	Not reported	Not reported
Rosenberg (2010) [46]	Exercise (Nintendo Wii gaming)	QIDS	Post-treatment (12 weeks); 24 weeks FU	-2.7; p = .004; -4.07; p = .001	(24 weeks FU) 53%	Not reported
Scogin (1987) [47]	Cognitive bibliotherapy	HRSD/GDS/BDI	Post-treatment (4 weeks); 1 month FU (IG Only)	HRSD: CB -8.5 AC -2.5 CG +1.1; p < .05; GDS: CB -5.8 AC -0.6 CG 0.0; p < .05; BDI CB -3.4 AC -1.7 CG -0.7; n.s.; HRSD: -6.3; GDS: -5.2; BDI: -0.7; No change compared with post-treatment; p > .05	Not reported	Not reported
Scogin (1989, 1990) [48,49]	1.Behavioral bibliotherapy; 2.Cognitive bibliotherapy	HRSD/GDS	Post-treatment (4 weeks); 6 months FU (IG only); 2 years FU (IG combined)	HRSD: BB -8.1 CB -8.8 CG -0.5; p < .05; GDS: BB -2.7 CB -5.6 CG -0.5; p < .05; HRDS:BB -8.7 CB -7.4; GDS: BB -5.2 CB -6.8; No change compared with post-treatment; p > .05; HRDS: -0.7; GDS -3.2; HRDS: no change compared with post-treatment; GDS: further improvement in bibliotherapy conditions (p < .05)	HRSD: IG 66% (completers only) CG 19%^e^	Not clearly reported
Serrano (2004) [50]	Life-review	CES-D	Post-treatment (8 weeks)	IG -10.25 and CG 0.0; p < .0001	Not reported	Not reported
Singh (1997) [51]	High intensity progressive resistance training	BDI/HRSD/GDS	Post-treatment (10 weeks)	BDI-: IG -11.5 and CG -4.6; p = .002;HRSD: IG -7.0 and CG -2.5; p = .008; GDS: IG -8.3 and CG -1.9; p = .0004	HRDS: IG 59% and CG 26%; p = .067	n.s.^f^
Thompson (1987) [52]	1. Cognitive therapy (CT); 2. Behavioral therapy (BT); 3. Brief Psychodynamic therapy (BPT)	BDI/HRSD; Diagnostic status (SADS-change)	Mid-treatment (6 weeks); Post-treatment (16 weeks)	BDI: IG (combined) -6.1 CG +1.2; p < .001; HRSD: IG (combined) -5.1 CG -0.3; p < .001; BDI: CT -11.7 BT -10.1 BPT -9.2; n.s.; HRSD: CT -8.7 BT -10.4 BPT -9.0; n.s.	Not reported	(Post-treatment) CT 52%, BT 57% BPT 47%; n.s.^g^
Titov (2015) [53]	iCBT	PHQ-9	Post-treatment (8 weeks); 3 months FU (IG only); 12 months FU (IG only)	IG -9.46 and CG -0.25; p < .001; -8.05; no change compared with post-treatment; -8.02; no change compared with post-treatment	IG 68.7% and CG 5.8%; p < .001^h^	IG 68.7% and CG 0%; p < .001^i^
Wuthrich (2013) [54]	CBT	GDS/CES-D	Post-treatment (12 weeks); 3 months FU (IG only)	GDS: IG -8.93 CG -1.97; p = .004; CES-D: IG -13.03 CG -1.45; p = .007; GDS: -8.3; CES-D -12.98; GDS and CES-D: no change compared with post-treatment	Unclear	Not reported for depression separately

AC, Attention Control; BDI, Beck Depression Inventory; CBT, Cognitive Behavior Therapy; CES-D, Center for Epidemiologic Studies Depression Scale; CG, Control Group; FU, Follow-Up; GDS, Geriatric Depression Scale; HAM-D Hamilton Rating Scale for Depression; HRSD, Hamilton Rating Scale for Depression; iCBT, Individual Cognitive Behavior Therapy; IG, Intervention Group; MADRS, Montgomery Asberg Depression Rating Scale; n.s., not significant; PFE, Physical Fitness Exercise; PHQ-9, Patient Health Questionnaire; PST, Problem Solving Therapy; QIDS, Quick Inventory of Depressive Symptomatology; SADS, Schedule for Affective Disorders and Schizophrenia.

* Difference between baseline and follow-up measurements;

**Defined as a ≥50% reduction in outcome measures, unless stated otherwise

^a^ Defined as a HSCL-20 score <0.5;

^b^ Defined as a reduction of the HRSD ≤11 and no longer having a major depressive episode, or as a HRDS <10;

^c^ Defined as the absence of depressive symptoms;

^d^ Remission defined as a MADRS score <7;

^e^ Defined as scores outside the range of the dysfunctional population, and a change according to the reliable change index;

^f^ Defined as change in diagnostic category;

^g^ Defined as scores outside the range of the dysfunctional population, and scores with a reliable change from Time 1;

^h^ Defined as a >5.20 reduction on the PHQ-9;

^i^ Defined as reliable improvement and a score below the clinical cut-off (PHQ-9 <10)

#### Cognitive behavioral therapy

Three RCTs [39,53,54] studied the effect of CBT on depressive symptoms. One RCT demonstrated that CBT group therapy was more effective than remaining on a waiting list, but found no difference between the effects of CBT and exercise [39]. Another RCT showed that, after treatment, CBT group therapy was effective at reducing depressive symptoms among participants suffering from depression with comorbid anxiety [54]. Individual CBT delivered through the internet was also more effective at reducing depressive symptoms after treatment than care as usual [53]. In the latter RCT, this effect was maintained at 3 months’ follow-up, although this was not compared to a control condition. In summary, individual CBT tended to be an effective treatment for reducing depressive symptoms compared with inactive control conditions among older adults, but the risk of bias ranged from low to moderate in the included studies.

#### Bibliotherapy

Four RCTs investigated the effect of individual bibliotherapy [37,44,47,48], and had low to moderate risk of bias. All RCTs showed that bibliotherapy was effective at reducing depressive symptoms at 4 weeks’ follow-up compared with remaining on a waiting list and being given a control form of bibliotherapy.

#### Life-review

All three RCTs investigating the effect of individual life-review on depression in older adults found a positive effect on depressive symptoms from 2 to 8 weeks’ follow-up [35,45,50]. One RCT [45] also reported a further improvement of depressive symptoms at 3 months’ follow-up, but did not compare this with a control condition. The risk of bias did differ a little between the included studies, ranging from high to moderate.

#### Exercise

Three RCTs [39,43,51] investigated the effect of exercise on depressive symptoms, with risk of bias assessments ranging from high to moderate. Compared with an active control group, one RCT did demonstrate an effect of exercise on depressive symptoms [51]. However, although two other RCTs did find that exercise had an effect compared with inactivity, no difference was observed between the exercise group and the active controls in each study (e.g., CBT [39] and social contact control [43]). In addition, one cohort study [46] studied the effect of exercise on depressive symptoms. It was not reported whether the intervention was delivered as group or as individual therapy, but showed reduced depression scores at 12 and 24 weeks’ follow-up compared with baseline.

#### Problem-solving therapy

Three RCTs studied the effect of individually delivered PST [36,41,42]. They all demonstrated that PST reduced depressive symptoms. Two of these RCTs [36,42] delivered PST in community agencies, and one combined PST with engagement in social activities [36]. The risk of bias varied from low to moderate.

#### Other

One RCT investigated the effect of receiving postcards on depressive symptomatology [55], but showed no effect at follow-up. Another RCT showed that three interventions (cognitive therapy, behavioral therapy, and brief psychodynamic therapy) had beneficial effects compared with controls (waiting list) [52] at 6 weeks (mid-treatment), but showed no differences between these three interventions after treatment (16 weeks). However, the effects of the three interventions were not compared with the control group after treatment, because those on the waiting list had started treatment.

## Discussion

### Summary of main findings

Of the five treatments studied in primary care, a meta-analysis on CBT yielded a significant result, indicating its potential benefit in primary care settings. There was also a positive effect with bright-light therapy, and although this is promising, it needs replication in a second trial in primary care before recommendations for implementation can be made. Unfortunately, we did not find convincing evidence in favor of exercise, PST, or behavioral activation for the treatment of depressive symptomatology in primary care, but better quality research is needed before we can reach any definitive conclusions. In addition, community-based studies showed promising short-term results for bibliotherapy, life-review, PST, behavioral therapy, brief psychodynamic therapy, and cognitive therapy, which might, therefore, be suitable for use as treatment strategies in primary care.

### Comparison with existing literature

Previous systematic reviews and meta-analyses focusing on the use of non-pharmacological treatment for depression in older patients have reported different findings to those in our review [17–20]. Two recent systematic reviews [18,20], for example, concluded that psychological treatments may be feasible for late-life depression (65+), but they did not perform formal meta-analyses. However, both of these reviews questioned the generalizability and efficacy because of the wide diversity of interventions, the low number of studies per intervention, and the poor quality of studies included. Moreover, neither review was limited to the primary care setting, and studies were excluded if they had a low quality assessment, leading to the exclusion of 73.9% [18] and 36.4% [20] of the identified studies, respectively. To be more comprehensive, we decided not to restrict ourselves to RCTs and not to exclude studies based on the quality assessment. This not only ensured that we could summarize all available evidence but also enabled us to formulate explicit targets for future research, such as instances where an included study was of poor quality but focused on a promising intervention.

Another two reviews included formal meta-analyses of the research [17,19], and they indicated that psychological treatments were moderately effective in the treatment of late-life depression. Specifically, one showed that CBT, life-review, and PST [17] were effective, while the other showed that CBT was more effective than a non-active control group [19]. However, these meta-analyses included studies conducted in clinical settings and with middle-aged participants (50/55+). These differences might explain why we could not replicate the finding that PST was an effective treatment for late-life depression in primary care; also, it should be noted that life-review therapy has been studied as treatment for late-life depression in primary care to date. Nonetheless, we confirmed the positive results for life-review and PST on depressive symptoms in community settings. We could also replicate the finding that CBT was an effective treatment modality for late-life depression at 6 months’ follow-up, though with a small effect size (SMD -0.21 [-0.40 to -0.03]) comparable to that reported in one of the previous studies [17]. The other meta-analysis demonstrated a much larger effect size (-1.35) when CBT was compared with inactive controls, but did not find an effect when comparing CBT to active controls [19]. The fact that we analyzed the effect of CBT compared with both active and inactive controls might explain this difference. Although one might question the clinical relevance of this small effect of CBT, it might be partly caused by a floor-effect of treatment associated with milder forms of depression as seen and treated within primary care.

Another systematic review found that physical exercise may be effective for late-life depression [56]. We could not replicate this finding, irrespective of the quality assessments of these studies, but it should be noted that the previously conducted review included studies recruiting non-depressed adults, and that none of the studies included in the earlier review [56] was conducted in a primary care setting.

Several differences can be seen when comparing the studies conducted in primary care with those conducted in the community. First, although more treatment modalities have been studied in community settings, it is questionable whether these treatment modalities are applicable in general practice. For example, creating a life-story book with personalized pictures [35] is overly time-consuming for most GPs or practice nurses. Second, the follow-up periods of the community-based studies were shorter than those conducted in primary care. Because none of the studies included a control condition beyond the assessment when treatment ended, no data is available on the sustainability of the effects. Third, most of the community studies only included self-referred participants, thereby introducing selection bias. Self-referred participants show the initiative to seek out interventions targeting depression, whereas in general, depressed older adults are more likely to be reluctant to seek help [57]. This purported selection bias might also explain some of the low percentages lost to follow-up in the self-referral studies performed in the community. Although it is conceivable that community-based interventions would also be effective in primary care, further research is needed to confirm this assumption. Finally, among the therapist-guided interventions, almost half were delivered by a postgraduate therapist or clinical psychologist in the community studies, while only one-third included a psychologist in the primary care studies. Because it is questionable whether clinical psychology services could be successfully embedded in general practice, due for example to higher costs for patients and/or insurances, future research should determine whether these interventions can be successfully given by a practice nurse or other allied healthcare professionals.

Several non-pharmacological treatments for late-life depression studied a community setting seem promising for implementation in primary care. First of all, PST demonstrated a beneficial effect in the community [36,41,42], but the only RCT conducted in primary care demonstrated no effect on depressive symptoms [34]. However, the risk of bias was lower in two of the community studies [36,42] compared with the study conducted in primary care [34], and among middle-aged adults the effectiveness of PST in primary care has been confirmed [58]. Due to the positive results of PST in the community setting and among middle-aged adults, we recommend a second RCT in primary care focusing on PST with a longer follow-up duration than the study included in our review (11 weeks) [34]. Moreover, the control group in this primary care study [34] existed of paroxetine or a placebo, while an attention control form of therapy would have been more adequate. Furthermore, bibliotherapy [37,44,47,48] and life-review [35,45,50] have demonstrated beneficial effects in community settings, although follow-up duration was short (maximum of 8 weeks). Before implementation in primary care, life-review and bibliotherapy need to be studied among primary care patients with a longer follow-up duration. Furthermore, these studies had some issues regarding their risk of bias, with a high risk of bias for the bibliotherapy studies [37,44,47,48] and a moderate risk of bias for two of the life-review studies [35,45], and these concerns need to be addressed in a future RCT conducted in primary care. Finally, the effect of behavioral activation therapy seems promising in a pilot cohort study conducted in primary care [26], and is currently being investigated in a well-designed RCT in primary care [59]. In addition to this latter RCT, also PST, bibliotherapy, and life-review should be studied in a RCT in primary care among depressed patients confirmed by a diagnostic interview and with at least a one-year follow-up.

### Limitations

First, although we decided to review the results narratively, we did diverge from the published protocol to perform a meta-analysis concerning the effect of CBT in primary care. This was because most of the included studies in primary care focused on CBT and the combined results of the individual studies were inconclusive. Although only two of the five individual studies indicated a beneficial effect of CBT, the meta-analysis confirmed a small but beneficial effect. Too few studies focusing on other non-pharmacological treatment options were conducted to perform a meta-analysis for these interventions; for example, two studies focused on exercise and both concluded that it was ineffective at follow-up, whereas only single studies were conducted for the other treatment modalities. Since we aimed to present an overview of the evidence for non-pharmacological treatments for late-life depression within primary care, we decided not to perform meta-analyses of studies conducted in the community, but to narratively review these studies in order to identify promising non-pharmacological treatments. Second, one of the search terms was “general practice OR synonym,” so we only found a few studies that were conducted in the community in the primary search. Although these settings were not the focus of our review, we wanted to include all studies that focused on non-pharmacological treatment options in primary care. Due to careful selection of studies from previous systematic reviews and meta-analyses, we could find and included more studies conducted in a community (n = 15) setting, consistent with the aim of our review (Fig 1). However, we cannot ignore the possibility that we did not include all studies focusing on non-pharmacological interventions for late-life depression conducted in a community setting. Third, included studies differed in their depression inclusion criterion, which may have introduced heterogeneity and thus may have affected the results of this review. However, the observed heterogeneity in our meta-analysis was small (I^2^ = 0%). Finally, limitations of included studies should also be acknowledged; such as the low number of included participants in primary care studies and the short follow-up period in community studies.

### Conclusion

Through this systematic review and meta-analysis, we aimed to provide general practitioners with a comprehensive summary of the available evidence for non-pharmacological treatments in late-life depression in primary care. We found a limited amount of studies studying a wide variety of non-pharmacological interventions. Moreover, these studies differed in their definition of depression, definition of remission, and follow-up duration. Although this limits the evidence for specific interventions, it does give merit for several promising therapeutic options for treatment of late-life depression within primary care. CBT was the only treatment option meeting the highest level of evidence according to the GRADE (Grading of Recommendations Assessment, Development and Evaluation) criteria, with a small but beneficial effect after meta-analysis. However, a wealth of alternative options were identified that could be delivered by well-trained nurses based on evidence that exists from studies in a community setting. This review indicates that bibliotherapy, life-review, PST, and behavioral activation therapy are the options most likely to be of benefit in primary care settings, but the paucity of high-quality research means that we can only conclude that these options warrant further investigation in RCTs performed in primary care.

## Supporting information

S1 Appendix Search strategy for different databases. Search strings for each part were combined using the “AND” Boolean statement.
(DOCX)

S1 PRISMA Checklist PRISMA checklist. (PDF)
