# Supplementary material for: Non-pharmacological treatment for depressed older patients in primary care: A systematic review and meta-analysis
Source: PLoS One. 2017 Sep 22;12(9):e0184666. doi: 10.1371/journal.pone.0184666 (PMC5609744; doi:10.1371/journal.pone.0184666)
Supplement: S1 Appendix — Search strings for each part were combined using the “AND” Boolean statement. (DOCX) [file pone.0184666.s001.docx]

**S1 Appendix. Search strategy for different databases.**

|  | Medline/Pubmed | PsychInfo | Cochrane/Central |
| --- | --- | --- | --- |
| Primary care | "General Practice"[Mesh] OR "Primary Health Care"[Mesh] OR "General Practitioners"[Mesh] OR "Family Practice"[Mesh] OR primary health care[tw] OR primary care[tw] OR general practi*[tw] OR general medicine[tw] OR general physician*[tw] OR general doctor*[tw] OR family doctor*[tw] OR family physician*[tw] OR family practi*[tw] OR family medicine[tw] | DE "General Practitioners" OR DE "Family Medicine" OR DE "Family Physicians" OR DE "Primary Health Care" OR primary health care OR primary care OR general practi* OR general medicine OR general physician* OR general doctor* OR family doctor* OR family physician* OR family practi* OR family medicine | primary health care or primary care or general practi* or general medicine or general physician* or general doctor* or family doctor* or family physician* or family practi* or family medicine |
| Aged | "Aged"[Mesh] OR "Geriatric Psychiatry"[Mesh] OR old*[tw] OR aged[tw] OR agin*[tw] OR agein*[tw] OR elderly[tw] OR late-life[tw] OR geriatric[tw] | DE "Aging" OR DE "Geriatric Psychiatry" OR DE "Geriatrics" OR old* OR aged OR agin* OR agein* OR elderly OR late life OR late-life OR geriatric | aged or old* or agin* or agein* or elderly or late-life or geriatric |
| Depression | "Depressive Disorder"[Mesh] OR "Depression"[Mesh] OR depress*[tw] | DE "Depression (Emotion)" OR DE "Major Depression" OR depress* | depress* |
| Treatment | “Psychotherapy”[MeSH] OR “Counseling”[MeSH] OR "running"[MeSH]) OR behavioral therap*[tw] OR behavioural therapy*[tw] OR behaviour therap*[tw] OR behavior therap*[tw] OR interpersonal therap*[tw] OR interpersonal psychotherap*[tw] OR cognitive therap*[tw] OR cognitive behavior*[tw] OR cognitive behaviour*[tw] OR psychotherap*[tw] OR counsel*[tw] OR problem-solving therap*[tw] OR problem solving therap*[tw] OR self-help[tw] OR activit*[tw] OR exercis*[tw] OR bibliotherap*[tw] OR running*[tw] OR life-review[tw] OR life review [tw] OR mindful*[tw] OR reminscence[tw] | DE "Psychotherapy" OR DE "Behavior Therapy" OR DE "Cognitive Behavior Therapy" OR DE "Cognitive Therapy" OR DE "Counseling" OR DE "Mindfulness" OR DE "Running" OR DE "Life Review" OR DE "Reminiscence" OR psychotherap* OR counsel* OR behavioral therap* OR behavioural therap* OR behavior therap* OR behaviour therap* OR interpersonal therap* OR interpersonal psychotherap* OR cognitive therap* OR cognitive behavior* OR cognitive behaviour* OR problem-solving therap* OR problem solving therap* OR self-help OR self help OR activit* OR exercis* OR running OR bibliotherap* OR life-review OR life review OR mindful* OR reminiscence | behavioural therap* or behaviour therap* or behavioral therap* or behavior therap* or interpersonal therap* or interpersonal psychotherap* or cognitive therap* or cognitive behavior* or cognitive behaviour* or psychotherap* or counsel* or problem-solving therap* or problem solving therap* or self-help or activit* or exercis* or bibliotherap* or reminiscence or running or life-review or life review or mindful* |

Search strings for each part were combined using the “AND” Boolean statement.
